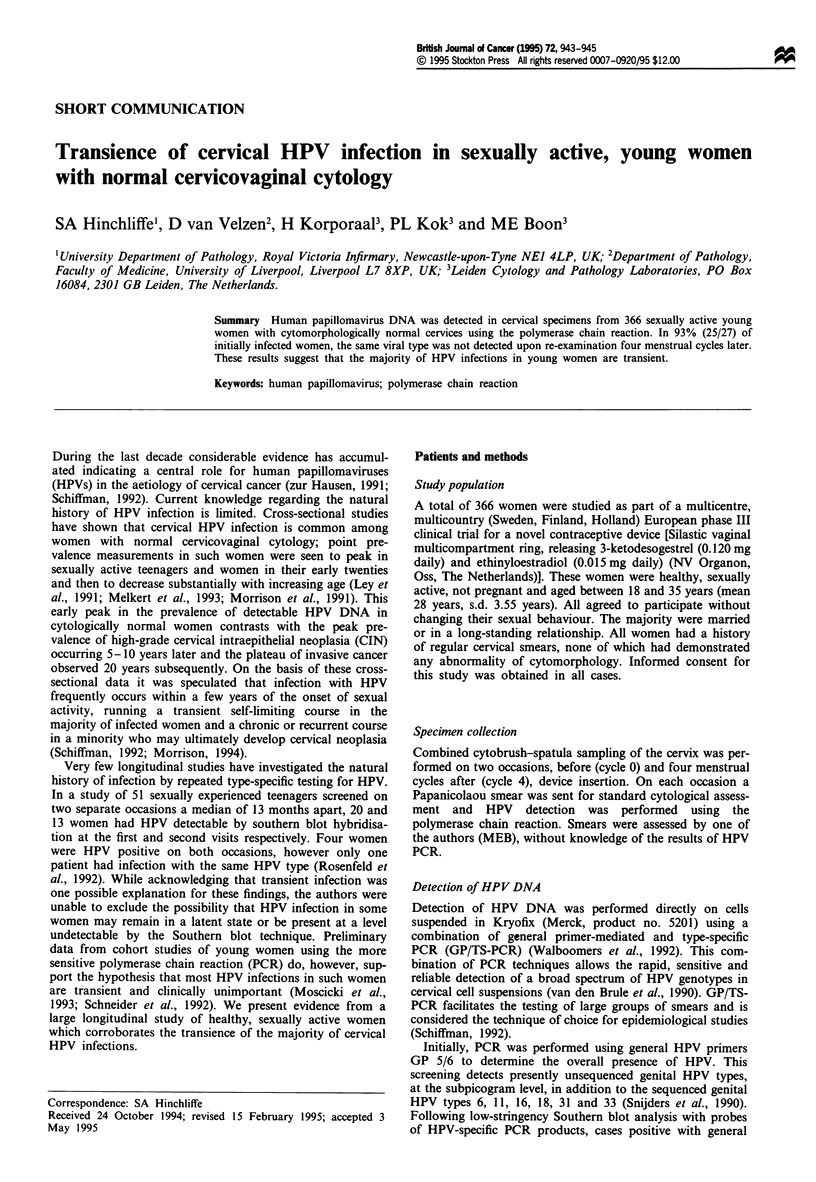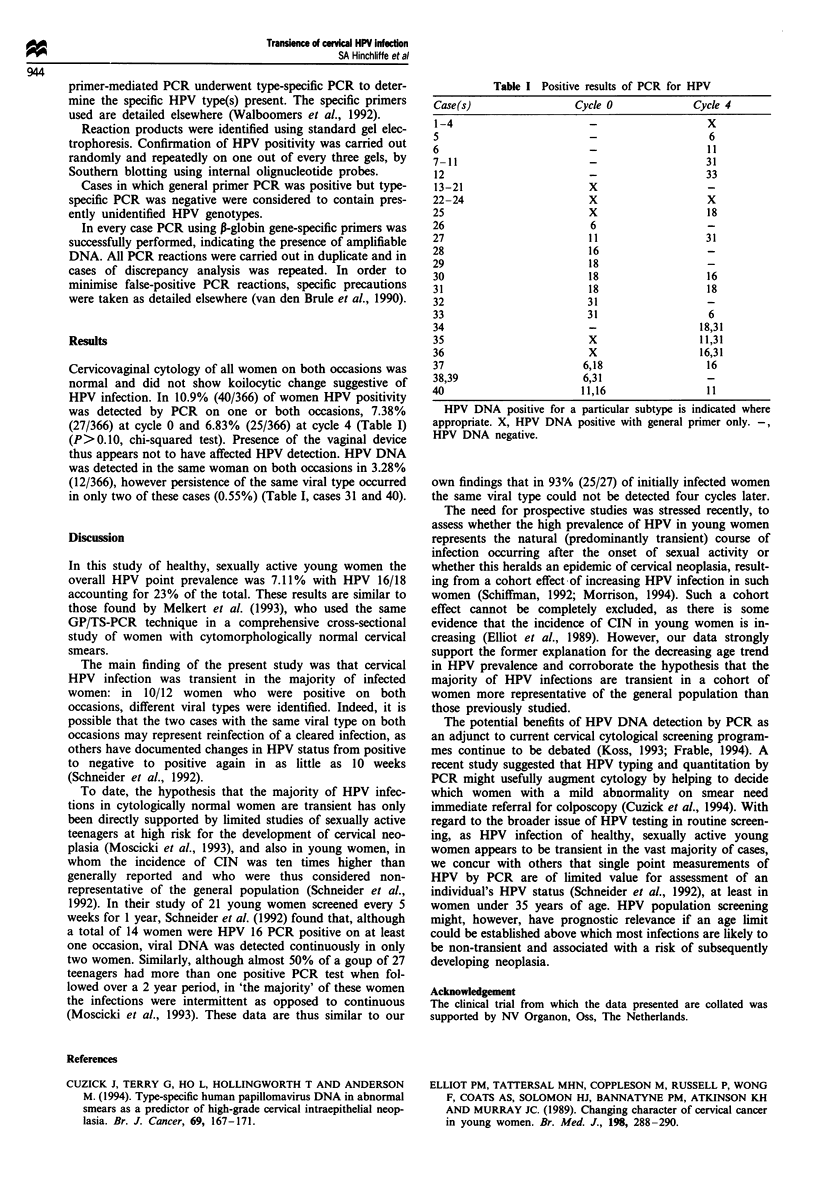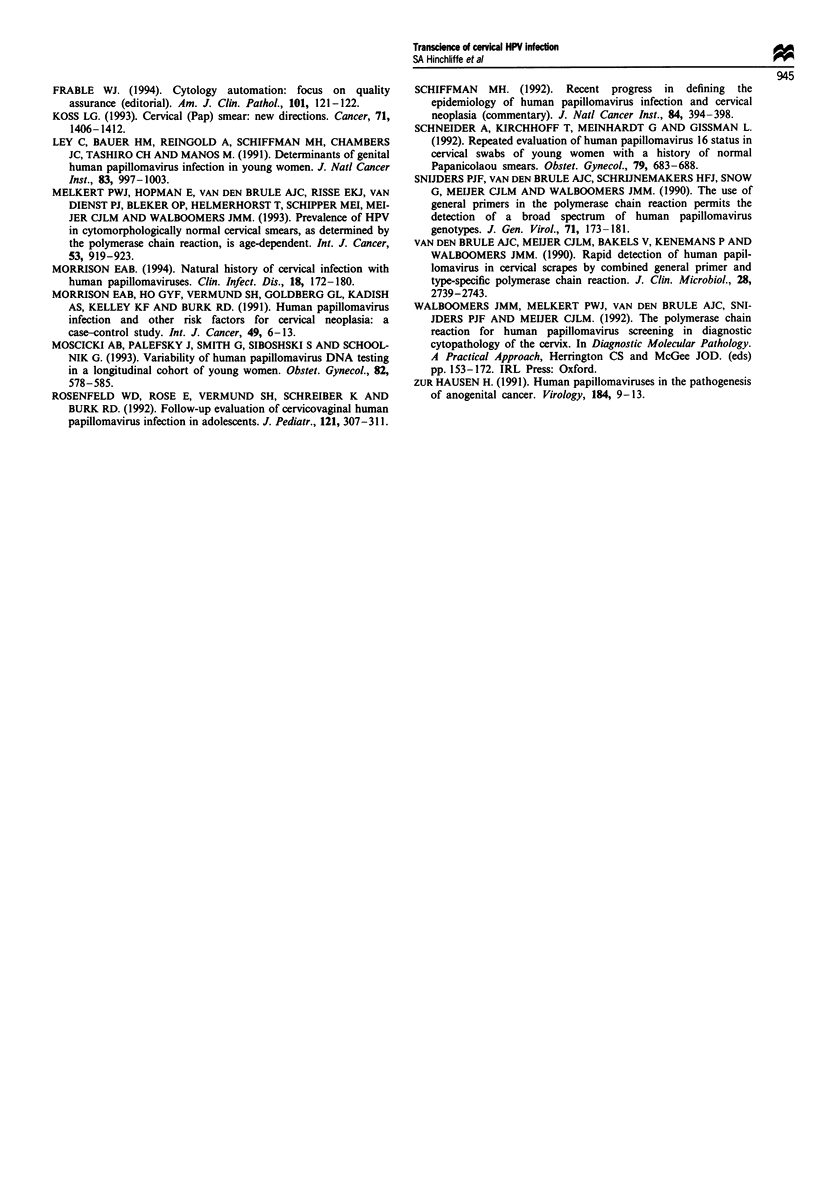# Transience of cervical HPV infection in sexually active, young women with normal cervicovaginal cytology.

**DOI:** 10.1038/bjc.1995.438

**Published:** 1995-10

**Authors:** S. A. Hinchliffe, D. van Velzen, H. Korporaal, P. L. Kok, M. E. Boon

**Affiliations:** University Department of Pathology, Royal Victoria Infirmary, Newcastle-upon-Tyne, UK.

## Abstract

Human papillomavirus DNA was detected in cervical specimens from 366 sexually active young women with cytomorphologically normal cervices using the polymerase chain reaction. In 93% (25/27) of initially infected women, the same viral type was not detected upon re-examination four menstrual cycles later. These results suggest that the majority of HPV infections in young women are transient.


British Journal of Cancer (1995) 72, 943-945
© 1995 Stockton Press All rights reserved 0007-0920/95 $12.00

SHORT COMMUNICATION

### Transience of cervical HPV infection in sexually active, young women with normal cervicovaginal cytology

SA Hinchliffe[1], D van Velzen[2], H Korporaal[3], PL Kok[3] and ME Boon[3]

[1]*University Department of Pathology, Royal Victoria Infirmary, Newcastle-upon-Tyne NE1 4LP, UK;* [2]*Department of Pathology, Faculty of Medicine, University of Liverpool, Liverpool L7 8XP, UK;* [3]*Leiden Cytology and Pathology Laboratories, PO Box 16084, 2301 GB Leiden, The Netherlands.*

**Summary** Human papillomavirus DNA was detected in cervical specimens from 366 sexually active young women with cytomorphologically normal cervices using the polymerase chain reaction. In 93% (25/27) of initially infected women, the same viral type was not detected upon re-examination four menstrual cycles later. These results suggest that the majority of HPV infections in young women are transient.

**Keywords:** human papillomavirus; polymerase chain reaction

During the last decade considerable evidence has accumulated indicating a central role for human papillomaviruses (HPVs) in the aetiology of cervical cancer (zur Hausen, 1991; Schiffman, 1992). Current knowledge regarding the natural history of HPV infection is limited. Cross-sectional studies have shown that cervical HPV infection is common among women with normal cervicovaginal cytology; point prevalence measurements in such women were seen to peak in sexually active teenagers and women in their early twenties and then to decrease substantially with increasing age (Ley *et al.*, 1991; Melkert *et al.*, 1993; Morrison *et al.*, 1991). This early peak in the prevalence of detectable HPV DNA in cytologically normal women contrasts with the peak prevalence of high-grade cervical intraepithelial neoplasia (CIN) occurring 5-10 years later and the plateau of invasive cancer observed 20 years subsequently. On the basis of these cross-sectional data it was speculated that infection with HPV frequently occurs within a few years of the onset of sexual activity, running a transient self-limiting course in the majority of infected women and a chronic or recurrent course in a minority who may ultimately develop cervical neoplasia (Schiffman, 1992; Morrison, 1994).

Very few longitudinal studies have investigated the natural history of infection by repeated type-specific testing for HPV. In a study of 51 sexually experienced teenagers screened on two separate occasions a median of 13 months apart, 20 and 13 women had HPV detectable by southern blot hybridisation at the first and second visits respectively. Four women were HPV positive on both occasions, however only one patient had infection with the same HPV type (Rosenfeld *et al.*, 1992). While acknowledging that transient infection was one possible explanation for these findings, the authors were unable to exclude the possibility that HPV infection in some women may remain in a latent state or be present at a level undetectable by the Southern blot technique. Preliminary data from cohort studies of young women using the more sensitive polymerase chain reaction (PCR) do, however, support the hypothesis that most HPV infections in such women are transient and clinically unimportant (Moscicki *et al.*, 1993; Schneider *et al.*, 1992). We present evidence from a large longitudinal study of healthy, sexually active women which corroborates the transience of the majority of cervical HPV infections.

Correspondence: SA Hinchliffe
Received 24 October 1994; revised 15 February 1995; accepted 3 May 1995

### Patients and methods

### *Study population*

A total of 366 women were studied as part of a multicentre, multicountry (Sweden, Finland, Holland) European phase III clinical trial for a novel contraceptive device [Silastic vaginal multicompartment ring, releasing 3-ketodesogestrel (0.120 mg daily) and ethinyloestradiol (0.015 mg daily) (NV Organon, Oss, The Netherlands)]. These women were healthy, sexually active, not pregnant and aged between 18 and 35 years (mean 28 years, s.d. 3.55 years). All agreed to participate without changing their sexual behaviour. The majority were married or in a long-standing relationship. All women had a history of regular cervical smears, none of which had demonstrated any abnormality of cytomorphology. Informed consent for this study was obtained in all cases.

### *Specimen collection*

Combined cytobrush–spatula sampling of the cervix was performed on two occasions, before (cycle 0) and four menstrual cycles after (cycle 4), device insertion. On each occasion a Papanicolaou smear was sent for standard cytological assessment and HPV detection was performed using the polymerase chain reaction. Smears were assessed by one of the authors (MEB), without knowledge of the results of HPV PCR.

### *Detection of HPV DNA*

Detection of HPV DNA was performed directly on cells suspended in Kryofix (Merck, product no. 5201) using a combination of general primer-mediated and type-specific PCR (GP/TS-PCR) (Walboomers *et al.*, 1992). This combination of PCR techniques allows the rapid, sensitive and reliable detection of a broad spectrum of HPV genotypes in cervical cell suspensions (van den Brule *et al.*, 1990). GP/TS-PCR facilitates the testing of large groups of smears and is considered the technique of choice for epidemiological studies (Schiffman, 1992).

Initially, PCR was performed using general HPV primers GP 5/6 to determine the overall presence of HPV. This screening detects presently unsequenced genital HPV types, at the subpicogram level, in addition to the sequenced genital HPV types 6, 11, 16, 18, 31 and 33 (Snijders *et al.*, 1990). Following low-stringency Southern blot analysis with probes of HPV-specific PCR products, cases positive with general primer-mediated PCR underwent type-specific PCR to determine the specific HPV type(s) present. The specific primers used are detailed elsewhere (Walboomers *et al.*, 1992).

Reaction products were identified using standard gel electrophoresis. Confirmation of HPV positivity was carried out randomly and repeatedly on one out of every three gels, by Southern blotting using internal olignucleotide probes.

Cases in which general primer PCR was positive but type-specific PCR was negative were considered to contain presently unidentified HPV genotypes.

In every case PCR using $\beta$-globin gene-specific primers was successfully performed, indicating the presence of amplifiable DNA. All PCR reactions were carried out in duplicate and in cases of discrepancy analysis was repeated. In order to minimise false-positive PCR reactions, specific precautions were taken as detailed elsewhere (van den Brule *et al.*, 1990).

### Results

Cervicovaginal cytology of all women on both occasions was normal and did not show koilocytic change suggestive of HPV infection. In 10.9% (40/366) of women HPV positivity was detected by PCR on one or both occasions, 7.38% (27/366) at cycle 0 and 6.83% (25/366) at cycle 4 (Table I) ($P > 0.10$, chi-squared test). Presence of the vaginal device thus appears not to have affected HPV detection. HPV DNA was detected in the same woman on both occasions in 3.28% (12/366), however persistence of the same viral type occurred in only two of these cases (0.55%) (Table I, cases 31 and 40).

**Table I** Positive results of PCR for HPV

| *Case(s)* | *Cycle 0* | *Cycle 4* |
|---|---|---|
| 1–4 | – | X |
| 5 | – | 6 |
| 6 | – | 11 |
| 7–11 | – | 31 |
| 12 | – | 33 |
| 13–21 | X | – |
| 22–24 | X | X |
| 25 | X | 18 |
| 26 | 6 | – |
| 27 | 11 | 31 |
| 28 | 16 | – |
| 29 | 18 | – |
| 30 | 18 | 16 |
| 31 | 18 | 18 |
| 32 | 31 | – |
| 33 | 31 | 6 |
| 34 | – | 18,31 |
| 35 | X | 11,31 |
| 36 | X | 16,31 |
| 37 | 6,18 | 16 |
| 38,39 | 6,31 | – |
| 40 | 11,16 | 11 |

HPV DNA positive for a particular subtype is indicated where appropriate. X, HPV DNA positive with general primer only. –, HPV DNA negative.

### Discussion

In this study of healthy, sexually active young women the overall HPV point prevalence was 7.11% with HPV 16/18 accounting for 23% of the total. These results are similar to those found by Melkert *et al.* (1993), who used the same GP/TS-PCR technique in a comprehensive cross-sectional study of women with cytomorphologically normal cervical smears.

The main finding of the present study was that cervical HPV infection was transient in the majority of infected women: in 10/12 women who were positive on both occasions, different viral types were identified. Indeed, it is possible that the two cases with the same viral type on both occasions may represent reinfection of a cleared infection, as others have documented changes in HPV status from positive to negative to positive again in as little as 10 weeks (Schneider *et al.*, 1992).

To date, the hypothesis that the majority of HPV infections in cytologically normal women are transient has only been directly supported by limited studies of sexually active teenagers at high risk for the development of cervical neoplasia (Moscicki *et al.*, 1993), and also in young women, in whom the incidence of CIN was ten times higher than generally reported and who were thus considered non-representative of the general population (Schneider *et al.*, 1992). In their study of 21 young women screened every 5 weeks for 1 year, Schneider *et al.* (1992) found that, although a total of 14 women were HPV 16 PCR positive on at least one occasion, viral DNA was detected continuously in only two women. Similarly, although almost 50% of a goup of 27 teenagers had more than one positive PCR test when followed over a 2 year period, in 'the majority' of these women the infections were intermittent as opposed to continuous (Moscicki *et al.*, 1993). These data are thus similar to our own findings that in 93% (25/27) of initially infected women the same viral type could not be detected four cycles later.

The need for prospective studies was stressed recently, to assess whether the high prevalence of HPV in young women represents the natural (predominantly transient) course of infection occurring after the onset of sexual activity or whether this heralds an epidemic of cervical neoplasia, resulting from a cohort effect of increasing HPV infection in such women (Schiffman, 1992; Morrison, 1994). Such a cohort effect cannot be completely excluded, as there is some evidence that the incidence of CIN in young women is increasing (Elliot *et al.*, 1989). However, our data strongly support the former explanation for the decreasing age trend in HPV prevalence and corroborate the hypothesis that the majority of HPV infections are transient in a cohort of women more representative of the general population than those previously studied.

The potential benefits of HPV DNA detection by PCR as an adjunct to current cervical cytological screening programmes continue to be debated (Koss, 1993; Frable, 1994). A recent study suggested that HPV typing and quantitation by PCR might usefully augment cytology by helping to decide which women with a mild abnormality on smear need immediate referral for colposcopy (Cuzick *et al.*, 1994). With regard to the broader issue of HPV testing in routine screening, as HPV infection of healthy, sexually active young women appears to be transient in the vast majority of cases, we concur with others that single point measurements of HPV by PCR are of limited value for assessment of an individual's HPV status (Schneider *et al.*, 1992), at least in women under 35 years of age. HPV population screening might, however, have prognostic relevance if an age limit could be established above which most infections are likely to be non-transient and associated with a risk of subsequently developing neoplasia.

**Acknowledgement**
The clinical trial from which the data presented are collated was supported by NV Organon, Oss, The Netherlands.

### References

CUZICK J, TERRY G, HO L, HOLLINGWORTH T AND ANDERSON M. (1994). Type-specific human papillomavirus DNA in abnormal smears as a predictor of high-grade cervical intraepithelial neoplasia. *Br. J. Cancer*, **69**, 167–171.

ELLIOT PM, TATTERSAL MHN, COPPLESON M, RUSSELL P, WONG F, COATS AS, SOLOMON HJ, BANNATYNE PM, ATKINSON KH AND MURRAY JC. (1989). Changing character of cervical cancer in young women. *Br. Med. J.*, **198**, 288–290.

FRABLE WJ. (1994). Cytology automation: focus on quality assurance (editorial). *Am. J. Clin. Pathol.*, **101**, 121-122.

KOSS LG. (1993). Cervical (Pap) smear: new directions. *Cancer*, **71**, 1406-1412.

LEY C, BAUER HM, REINGOLD A, SCHIFFMAN MH, CHAMBERS JC, TASHIRO CH AND MANOS M. (1991). Determinants of genital human papillomavirus infection in young women. *J. Natl Cancer Inst.*, **83**, 997-1003.

MELKERT PWJ, HOPMAN E, VAN DEN BRULE AJC, RISSE EKJ, VAN DIENST PJ, BLEKER OP, HELMERHORST T, SCHIPPER MEI, MEIJER CJLM AND WALBOOMERS JMM. (1993). Prevalence of HPV in cytomorphologically normal cervical smears, as determined by the polymerase chain reaction, is age-dependent. *Int. J. Cancer*, **53**, 919-923.

MORRISON EAB. (1994). Natural history of cervical infection with human papillomaviruses. *Clin. Infect. Dis.*, **18**, 172-180.

MORRISON EAB, HO GYF, VERMUND SH, GOLDBERG GL, KADISH AS, KELLEY KF AND BURK RD. (1991). Human papillomavirus infection and other risk factors for cervical neoplasia: a case-control study. *Int. J. Cancer*, **49**, 6-13.

MOSCICKI AB, PALEFSKY J, SMITH G, SIBOSHSKI S AND SCHOOLNIK G. (1993). Variability of human papillomavirus DNA testing in a longitudinal cohort of young women. *Obstet. Gynecol.*, **82**, 578-585.

ROSENFELD WD, ROSE E, VERMUND SH, SCHREIBER K AND BURK RD. (1992). Follow-up evaluation of cervicovaginal human papillomavirus infection in adolescents. *J. Pediatr.*, **121**, 307-311.

SCHIFFMAN MH. (1992). Recent progress in defining the epidemiology of human papillomavirus infection and cervical neoplasia (commentary). *J. Natl Cancer Inst.*, **84**, 394-398.

SCHNEIDER A, KIRCHHOFF T, MEINHARDT G AND GISSMAN L. (1992). Repeated evaluation of human papillomavirus 16 status in cervical swabs of young women with a history of normal Papanicolaou smears. *Obstet. Gynecol.*, **79**, 683-688.

SNIJDERS PJF, VAN DEN BRULE AJC, SCHRIJNEMAKERS HFJ, SNOW G, MEIJER CJLM AND WALBOOMERS JMM. (1990). The use of general primers in the polymerase chain reaction permits the detection of a broad spectrum of human papillomavirus genotypes. *J. Gen. Virol.*, **71**, 173-181.

VAN DEN BRULE AJC, MEIJER CJLM, BAKELS V, KENEMANS P AND WALBOOMERS JMM. (1990). Rapid detection of human papillomavirus in cervical scrapes by combined general primer and type-specific polymerase chain reaction. *J. Clin. Microbiol.*, **28**, 2739-2743.

WALBOOMERS JMM, MELKERT PWJ, VAN DEN BRULE AJC, SNIJDERS PJF AND MEIJER CJLM. (1992). The polymerase chain reaction for human papillomavirus screening in diagnostic cytopathology of the cervix. In *Diagnostic Molecular Pathology. A Practical Approach*, Herrington CS and McGee JOD. (eds) pp. 153-172. IRL Press: Oxford.

ZUR HAUSEN H. (1991). Human papillomaviruses in the pathogenesis of anogenital cancer. *Virology*, **184**, 9-13.